# Assessment of postoperative health functioning after knee arthroplasty in relation to pain catastrophizing: a 6-month follow-up cohort study

**DOI:** 10.7717/peerj.9903

**Published:** 2020-09-09

**Authors:** Marc Terradas-Monllor, Mirari Ochandorena-Acha, Julio Salinas-Chesa, Sergi Ramírez, Hector Beltran-Alacreu

**Affiliations:** 1 Faculty of Health Sciences and Welfare, University of Vic—Central University of Catalonia, Vic, Spain; 2 Research Group on Methodology, Methods, Models and Outcomes of Health and Social Sciences (M3O), Faculty of Health Sciences and Welfare, Center for Health and Social Care Research (CESS), University of Vic—Central University of Catalonia (UVIC-UCC), Vic, Spain; 3 Institut de Rehabilitació i Terapèutica Biofísica (IRITEB), Badalona, Spain; 4 Programa de Doctorat en Medicina i Recerca Translacional, Facultat de Medicina, Universitat de Barcelona, Barcelona, Spain; 5 Departamento de Fisioterapia, Centro Superior de Estudios Universitarios La Salle, Universidad Autónoma de Madrid, Madrid, Spain; 6 Motion in Brains Research Group, Instituto de Neurociencias y Ciencias del Movimiento, Centro Superior de Estudios Universitarios La Salle, Universidad Autónoma de Madrid, Madrid, Spain

**Keywords:** Knee arhtroplasty, Pain catastrophizing, Rehabilitation

## Abstract

**Background:**

Knee arthroplasty (KA) is a typically successful surgical procedure commonly performed to alleviate painin participants with end-stage knee osteoarthritis. Despite its beneficial effects, a significant proportion of individuals with KA continue experiencing persistent pain and functional limitations. The purpose of this study was to assess the postoperative outcomes after KA in relation to postoperative pain catastrophizing.

**Methods:**

Participants were recruited at a domiciliary physiotherapy service, using a prospective, observational, hypothesis-generating cohort design. Participants were divided into two groups based on their Pain Catastrophizing Scale (PCS) total score (50th percentile), which resulted in high and low PCS groups. The primary outcome measure was the Western Ontario and McMaster Universities Osteoarthritis Index (WOMAC). In addition, quality of life, walking speed, physical performance, range of motion, and pain were measured. Outcome measures were collected at baseline (1 week postoperatively) and at follow-up (1, 3, and 6 months postoperatively).

**Results:**

A total of 60 participants (21 total KA and 39 unicompartmental KA) were recruited. Individuals with a higher degree of pain catastrophizing showed significantly higher WOMAC total scores at every follow-up, indicating poorer health functioning (*p* < 0.01). Similarly, the high PCS group showed higher WOMAC pain, stiffness and disability subscale scores (*p* < 0.05), poorer quality of life (*p* < 0.01), and poorer physical performance (*p* < 0.05) at every follow-up. In addition, the high PCS group achieved a slower walking speed at baseline and at 3 months follow-up (*p* < 0.05), and a higher degree of pain at rest, on walking and on knee flexion at every follow-up (*p* < 0.01, *p* < 0.05 and *p* < 0.05, respectively) except for walking pain at 3 months follow-up. No significant differences were observed between groups in range of motion, except for active knee extension at the 6-month follow-up (*p* < 0.05). Effect size was large at 1 month follow-up in WOMAC total score (*r* = 0.578) and pain intensity during knee flexion (*r* = 0.529). Longitudinal analyses revealed different improvement trends during the rehabilitation process between groups, with a lack of significant improvements in the high PCS group between the 3- and 6-month follow-up in WOMAC total score, WOMAC pain, WOMAC disability, quality of life, physical performance, active knee extension and resting pain (*p* > 0.05).

**Conclusion:**

The results of the present study suggest that participants with high postoperative pain catastrophizing might have poorer outcomes during the rehabilitation process after KA. Future work should seek to clarify if this relationship is causal.

## Introduction

### Background

Knee arthroplasty (KA) is a commonly performed surgical procedure designed to alleviate knee pain and improve function in individuals with severe, end-stage knee osteoarthritis (OA) when nonsurgical management is no longer effective (Lewis et al., 2015). Due to its success in relieving pain, reducing deformity, and improving function, KA has become one of the most widespread orthopedic surgeries (Witvrouw et al., 2009). Despite its evident beneficial effects, only one-third of participants report no functional problems following surgery (Wright et al., 2004). Approximately 20% report dissatisfaction with their functional ability a year or more after surgery (Jones et al., 2000), and between 13% and 30% of the patients continue to experience high levels of pain, disability, and a significant reduction in quality of life (Forsythe et al., 2008).

The study of risk factors for poor outcomes after KA is a fundamental step in designing perioperative interventions to improve outcomes (Wylde et al., 2017). Among all studied risk factors, some are considered nonmodifiable, such as sex and age. However, other risk factors might respond to specific interventions, such as pain catastrophizing, preoperative pain intensity, and mental health (Lewis et al., 2015).

The term “pain catastrophizing” has been used to describe a type of response to painful experiences that is likely to be associated with adverse pain outcomes (Sullivan, Bishop & Pivik, 1995). This response has emerged as one of the strongest independent predictors of poor outcomes after KA (Lewis et al., 2015). Pain catastrophizing is characterized by a tendency to focus excessively on pain sensations (e.g., rumination), to exaggerate the threat of pain sensations (e.g., magnification) and to perceive oneself as being unable to control pain symptoms (e.g., helplessness) (Sullivan et al., 2001). Furthermore, it is important to note that the effect of pain catastrophizing appears not to be influenced by the follow-up period, indicating that this factor continues to exert an effect months to years after surgery (Lewis et al., 2015).

Consequently, it appears that the identification of preoperative at-risk patients and specific interventions for them could improve their postoperative outcomes (Banka et al., 2015; Bierke & Petersen, 2017). In addition, given the strength of the association between psychosocial variables and postoperative outcomes after knee arthroplasty, the development and evaluation of interventions specifically designed to target psychosocial factors in those scheduled for a KA are justified (Sullivan et al., 2011). To this end, interventions explicitly targeting participants with high levels of preoperative pain catastrophizing have been evaluated, with no significant postoperative improvements (Riddle et al., 2019; Birch et al., 2020). Therefore, in addition to preoperative risk factors, early postsurgical factors might also limit rehabilitation and recovery and could be associated with poor outcomes (Wylde et al., 2017). There is evidence that some factors, such as self-efficacy, might be more strongly associated with outcomes when they are assessed in the postoperative period, rather than in the preoperative period (Van den Akker-Scheek et al., 2007). Furthermore, the evidence suggests that the prediction of persistent postsurgical pain is more accurate when both preoperative and postoperative risk factors have been assessed (Althaus et al., 2012).

Along these lines, early postoperative physical therapy interventions for participants with KA usually aim to improve outcomes such as range of motion (ROM), pain intensity and physical function, and they have shown little or no effect on long-term outcomes (Artz et al., 2015). This result could be because acute postoperative pain and functional limitations are not risk factors for poor outcomes after KA, or perhaps these interventions require evaluation in trials that are focused on high-risk participants. To evaluate the effect of early postoperative physiotherapy interventions on long-term outcomes after knee arthroplasty, more research is needed to identify postoperative patient-related risk factors (Wylde et al., 2017).

### Objectives

The primary aim of this study was to investigate the postoperative health functioning at 1, 3, and 6 months following KA in relation to postoperative pain catastrophizing. A second objective was to assess the health-related quality of life, pain, walking speed, physical performance, and range of motion in relation to postoperative pain catastrophizing. The hypothesis of the present study is that subjects with higher postoperative pain catastrophizing achieve worse postoperative outcomes during the rehabilitation period.

## Methods

### Study design

The study used a prospective, observational hypothesis-generating cohort design according to the Strengthening the Reporting of Observational Studies in Epidemiology (STROBE) (Von Elm et al., 2007). This study was conducted following the declaration of Helsinki. The study protocol received approval from The Research Ethics Committee of University of Vic—Central University of Catalonia (59/2018). The protocol was registered at *clinicaltrials.gov* (NCT03378440).

### Deviations from protocol

The purpose was altered after the planning of the study (clinicaltrials.gov: NCT03378440), which initially sought to identify preoperative risk factors for persistent postoperative pain after total knee arthroplasty. All protocol changes are listed below:

• November 2017—A prospective cohort study in collaboration with a university hospital is registered. This study aimed to investigate the relationship between preoperative psychosocial factors and postoperative pain and function after knee arthroplasty.
• December 2019—Collaboration between the researchers and the hospital ceases, and all gathered data is deleted. A new study in collaboration with a domiciliary rehabilitation provider company is registered using the same registration entry. The latest study aims to investigate the relationship between postoperative psychosocial factors and pain and function outcomes after knee arthroplasty.
• February 2020—Registration error adjustments. Variables such as pain catastrophizing, physical performance, or walking speed were being evaluated, but they were not listed in the registration. Therefore, these variables were added to the registry.

As a consequence of the protocol deviations described above, the number of possible participants was reduced. The authors decided to develop a first hypothesis-generating study with fewer participants than estimated. The present study also helped the authors deciding if this research line was worth it to be continued.

Initially, the original statistical analysis plan was to perform a multivariate regression analysis, which would include psychosocial variables such as pain-related fear of movement, anxiety, depression, or pain attitudes. However, due to the small sample size of the present study, the authors decided to omit several of the assessed variables, and use an alternative method to investigate whether, within this cohort, certain groups existed in the risk of delayed recovery.

Preoperative pain catastrophizing has been widely investigated. Nevertheless, its postoperative association with outcomes remains unclear. Besides, pain catastrophizing is considered of high research and clinical interest. For these reasons, it was selected as a grouping variable. This decision was made after data collection, but the hypothesis was established before the data analysis. Therefore, the authors were blinded to the results.

### Setting and data collection

Surgical interventions were performed in two public hospitals, *Germans Trias i Pujol Hospital* in Badalona, Barcelona (Spain), and *Hospital Foundation of the Holy Spirit* in Santa Coloma de Gramenet, Barcelona (Spain). The data collection period started in December 2018 and ended in January 2020. Measurements were obtained by three different physiotherapists, and each participant was assessed by the same physiotherapist. For physical measurements, all physiotherapists had a meeting prior to enrollment to establish common criteria and prevent biases.

### Participants

Eligible participants were women and men between 50 and 90 years of age with a total or unicompartmental KA due to primary OA. Exclusion criteria for the study were participants who underwent revision surgery; were operated due to secondary osteoarthritis; were unable to read or speak in Spanish; had a diagnosis of inflammatory arthritis or severe depression; and those admitted to the domiciliary physiotherapy service after the first assessment.

### Variables

#### Demographic and health data

Demographic and health data were recorded upon enrollment of the participants. They included age, sex, body mass index (BMI), Charlson comorbidity index (Charlson et al., 1987), smoking status, alcohol intake, type of surgery and educational level.

#### Health functioning associated with osteoarthritis

The Spanish version of the Western Ontario and McMaster Universities Osteoarthritis Index (WOMAC) was used as a measure of health functioning after KA (Escobar et al., 2002). The WOMAC is a multidimensional scale composed of 24 items grouped into three dimensions: pain (five items), stiffness (two items) and physical function (seventeen items). The WOMAC uses a 5-point Likert scale with responses ranging from 0 = none to 4 = extreme. The final score for the WOMAC was determined by summing the aggregate scores for pain, stiffness and physical function (Escobar et al., 2002). The WOMAC is valid and reliable for assessing health functioning in OA participants and is sensitive to changes in health functioning in those who underwent knee arthroplasty (Bellamy et al., 1988; Bellamy et al., 1992; Bellamy, 1989).

#### Pain catastrophizing

The Spanish version of the Pain Catastrophizing Scale (PCS) was used to assess thoughts and feelings related to pain experiences (García Campayo et al., 2008). The PCS is a 13-item self-administered questionnaire composed of three subscales: rumination, magnification and helplessness. The PCS uses a 5-point Likert scale with responses ranging from 0 = not at all to 4 = all the time. The Spanish version of the PCS has been shown to have high internal consistency (Cronbach’s alpha total = .79; rumination = .82; magnification = .72, helplessness = .80) (García Campayo et al., 2008) and to be associated with postsurgical persistent pain after knee arthroplasty (Lewis et al., 2015).

#### Health-related quality of life

The Euro Quality of Life 5D-5L (EQ-5D-5L) instrument was used to assess health-related quality of life (HRQoL) (Conner-Spady et al., 2015). The EQ-5D-5L consists of two pages: the first is based on a descriptive system that defines health in terms of five dimensions: mobility, self-care, usual activities, pain/discomfort and anxiety/depression. Each dimension subsequently has five response categories: no problems, slight problems, moderate problems, severe problems and extreme problems (Conner-Spady et al., 2015). A health status is composed of 5 scores, one score for each dimension, and a preference-based scoring function is used to convert the descriptive system to a summary index score (ranging from states “worse than dead” (<0) to full health (1)) (Van Hout et al., 2012). The second page has a 20 cm vertical visual analogue scale (VAS), in which the participants rated their actual health state on a scale from 0 (poorest imaginable health) to 100 (best imaginable health) (Conner-Spady et al., 2015). The EQ-5D-5L has been validated for the Spanish population (Hernandez et al., 2018), and has been shown to be more valid than its previous version (5D-3L) in participants with hip and knee osteoarthritis, especially in terms of the dimensions relevant to this population—mobility, daily activities, and pain/discomfort (Conner-Spady et al., 2015).

#### Pain

Pain was assessed using the scores on the WOMAC subscale of pain (WOMAC pain) (Escobar et al., 2002). In addition, a 100-mm VAS (0 = no pain, 100 = worst imaginable pain) was used separately to assess resting pain, walking pain and knee flexion pain (VAS rest, VAS walk and VAS flex, respectively) (Carlsson, 1983).

#### Walking speed

The 4MWT is a simple method to evaluate walking speed in individuals undergoing lower extremity surgery during inpatient rehabilitation. It requires little space and it can be used in almost any clinical environment. To perform the 4MWT, an 8-meter path was marked with the first and last 2 m reserved for acceleration and deceleration, respectively. The stopwatch was started as soon as the participants’ leg passed over the starting line and stopped when it passed over the 4-meter line. The test was performed three times and the final score was the mean of the three attempts. Before each attempt, we gave the same verbal instructions to the participants on how to perform the test: “walk as fast as possible in a comfortable way”. Assistive devices such as walkers or crutches were allowed. The 4MWT showed excellent test-retest reliability (Unver et al., 2017).

#### Physical performance

The 30-second Chair Stand Test (30 CST) is a reliable and valid measurement tool for lower extremity strength (Unver et al., 2015). Participants were asked to sit and stand as quickly and safely as possible in 30 s, using a standard chair with a seat height of 43 cm (Gill & McBurney, 2008). An inability to rise from a sitting position is recognized by the World Health Organization as a disabling condition. Adequate quadriceps muscle strength is necessary to perform this activity and is correlated with walking speed, independent ambulation, and stair climbing (Unver et al., 2015). The 30 CST showed excellent reliability in participants with KA (Unver et al., 2015).

#### Range of motion

The active and passive ROM were evaluated for both knee flexion and extension movements, using a universal goniometer. The universal goniometer is a valid and reliable measurement tool for knee ROM measurement (Brosseau et al., 2001).

### Outcome measurement

Prior to assessment, all participants provided verbal and written informed consent. The assessments were performed at the participant’s home and at multiple time points, including 1 week, 1 month, 3 months, and 6 months postoperatively. Most of the measurements are self-administered questionnaires and require no or minimal interaction with the assessor. Due to the advanced age of some of the participants, assessors provided support during the questionnaire completion if needed. In addition, all questionnaires were provided in a random order to prevent possible biases, such as automation of the responses due to fatigue.

### Study size

The recruitment of participants took place between December 2018 and July 2019 on a consecutive (non-random) basis in a domiciliary rehabilitation service. The number of subjects who were admitted for rehabilitation during the study period determined the sample size.

The cut-off for PCS was 14.5 (range 0–52) according to the 50th percentile on data collected from 60 consecutive participants at baseline. The baseline point was 1 week after surgery, just after being admitted for postoperative rehabilitation. The decision for the cut-off setting was based in the PCS—User Manual by Sullivan et al. (2009). Individuals who score between the 50th and 75th percentiles on the PCS are considered at moderate risk for the development of chronicity, and those who score above the 75th percentile are considered at high risk (Sullivan, Bishop & Pivik, 2009).

### Statistical analysis

All data were analyzed using Statistical Package for the Social Sciences (SPSS 26, SPSS Inc., Chicago, IL, USA). The normal distribution of all measures was assessed for the two groups using the Shapiro–Wilk test (*p* > 0.05). Independent t-tests and the Mann Whitney U test were used to compare the demographic and clinical characteristics of participants from the two groups. Categorical variables were compared by the chi-squared test for variables with two categories, and the linear chi-squared test for those with three or more categories.

Due to a lack of normal distribution for most variables and the small sample size, the authors used nonparametric statistics. First of all, the correlation between PCS and pain intensity was assessed using Spearman’s rank test. Psychosocial variables such as pain catastrophizing are highly influenced by pain itself, therefore their correlation should be assessed (Wade, Riddle & Thacker, 2012). Coefficients (*ρ*) between 0 and 0.40 were considered as weak correlation; values between 0.40–0.69 as moderate correlation; and values between 0.70–0.89 as strong correlation (Schober & Schwarte, 2018). Then, the Mann–Whitney U test was used to analyze the difference between groups at each follow-up. Also, the between-group effect size (*r*) of Mann–Whitney’s U test was calculated using the following formula: $r= \frac{Z}{\sqrt{N}} $, where *N* is the total of the samples. The values of *r* were considered small when they were higher than 0.1, medium when higher than 0.3 and large when higher than 0.5 (Field, 2005).

For the longitudinal analyses, Friedman’s ANOVA was employed to analyze changes in intragroup results, and the Wilcoxon signed rank test was performed for *post hoc* intragroup comparisons.

## Results

**Figure 1:**
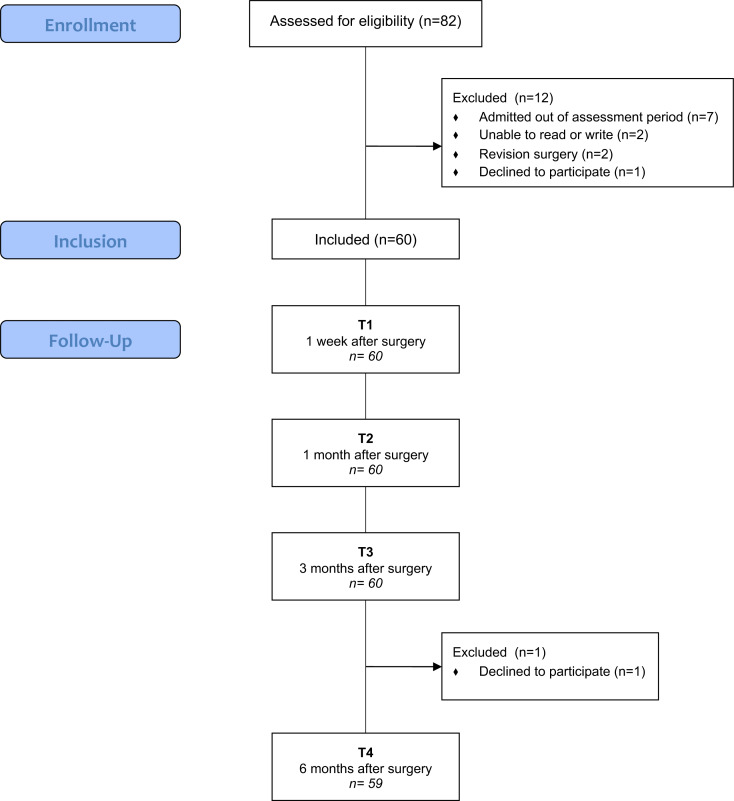
Flowchart of KA patients (screening, inclusion and assessment at all data points).

Eighty-two participants were assessed for eligibility, and 12 were excluded because they did not meet the inclusion criteria (*n* = 11) or declined to participate (*n* = 1). The remaining 60 participants were recruited and followed over a 6-month period. Only one participant withdrew at the final assessment. Figure 1 shows the flow chart for this study.

According to the cut-off score (≥14.5) for the 50th percentile of the 1-week PCS, participants were divided into two groups: high or low degree of pain catastrophizing. The demographic and health data on participants concerning pain catastrophizing are summarized in Table 1. There were no significant differences between groups at domiciliary physiotherapy admission in terms of age, sex, BMI, comorbidities, smoking status, alcohol intake, type of surgery, or education level (*p* > 0.05).

**Table 1:** Sociodemographic and health characteristics of participants.

**Population description**	**Low degree of PCS (<14.5)**	**High degree of PCS (≥14.5)**	***p* value (≤0.05)**
Age (SD)	70.17 (7.6)	70.30 (7.05)	0.947^a^
Sex			
Male, *n* (%)	10 (33.3%)	9 (30%)	0.781^b^
Female, *n* (%)	20 (66.7%)	21 (70%)	
Body mass index (BMI)	29.69 (4.39)	30.60 (4.35)	
Normal, *n* (%)	4 (13.3%)	3 (10%)	
Overweight, *n* (%)	13 (43.3%)	11 (36.7%)	0.598^c^
Type I Obesity, *n* (%)	8 (26.7%)	11 (36.7%)	
Type II Obesity, *n* (%)	4 (13.3%)	4 (13.3%)	
Type III Obesity, *n* (%)	1 (3.3%)	1 (3.3%)	
Charlson Comorbidity Index (CCI)			
1, *n* (%)	1 (3.3%)	0	
2, *n* (%)	9 (30%)	9 (30%)	
3, *n* (%)	10 (33.3%)	15 (50%)	0.582^c^
4, *n* (%)	8 (26.7%)	5 (16.7%)	
5, *n* (%)	1 (3.3%)	1 (3.3%)	
6, *n* (%)	1 (3.3%)	0	
Smoking			
Never smoked, *n* (%)	20 (66.7%)	21 (70%)	
Quit smoking, *n* (%)	9 (30%)	7 (23.3%)	1.000^c^
Smoker, *n* (%)	1 (3.3%)	2 (6.7%)	
Alcohol			
Never, *n* (%)	10 (33.3%)	13 (43.3%)	0.238^c^
Minimal consumption, *n* (%)	18 (60%)	17 (56.7%)	
Usual consumption, *n* (%)	2 (6.7%)	0	
Type of surgery			
TKA, *n* (%)	10 (33.3%)	11 (36.7%)	0.787^b^
UKA, *n* (%)	20 (66.7%)	19 (63.3%)	
Education level			
Read and write, *n* (%)	12 (40%)	13 (43.3%)	
Elementary, intermediate, *n* (%)	12 (40%)	10 (33.3%)	0.761^c^
Secondary, vocational, *n* (%)	6 (20%)	5 (16.7%)	
University, *n* (%)	0	2 (6.7%)	

**Notes.**

a Independent samples *t*-test.

b Pearson’s chi-squared test.

c Linear chi-squared test.

### Correlation analysis

Considering that the values of *ρ* require a moderate or strong correlation, when they are between 0.40–0.69 and 0.70–0.90 respectively (Schober & Schwarte, 2018), a moderate correlation was observed between PCS and WOMAC pain (*ρ* = 0.687, *p* < 0.01), VAS rest (*ρ* = 0.688, *p* < 0.01), VAS walking (*ρ* = 0.636, *p* < 0.01), and VAS flexion (*ρ* = 0.643, *p* < 0.01).

### Outcome measure regarding pain catastrophism

#### Between-group analysis

Participants with a higher degree of postsurgical pain catastrophism showed significantly poorer results compared with those with a lower degree (Table 2 and Figs. S1–S12). WOMAC total scores were significantly lower at each of the four assessment points in the low PCS group (*p* < 0.01), indicating better health functioning associated with OA. WOMAC pain, stiffness, and disability subscale scores were also significantly lower in the low PCS group at every follow-up. Regarding EQ-5D-5L scores, significantly higher scores were obtained in the low PCS group at each assessment point (*p* < 0.01), indicating better quality of life. When it comes to functional outcomes, the low PCS group showed a significantly higher walking speed at 1 week (*p* = 0.037) and 3 months (*p* = 0.032), but no statistically significant differences were observed at 1 and 6 months (*p* = 0.249 *and 0.06, respectively*). The low PCS group also showed better physical performance, achieving higher 30 CST scores at each time point (*p* = 0.05). No statistically significant differences were observed between the groups in any active or passive ROM measurements at any time point assessment, except for active knee extension at 6 months (*p* = 0.039). Finally, the high PCS group showed higher VAS pain levels at every follow-up at rest (*p* < 0.01), walking (*p* < 0.05) and knee flexion (*p* < 0.05), except for walking pain at 3 months (*p* = 0.053).

#### Between-group effect size

Large effect sizes at 1 week were obtained in WOMAC total scores (*r* = 0.563); WOMAC pain (*r* = 0.622); WOMAC disability (*r* = 0.519); EQ-5D-5L (*r* = 0.608); VAS pain at rest (*r* = 0.581), walking (*r* = 0.573) and knee flexion (*r* = 0.529). At the 1-month follow-up, WOMAC total (*r* = 0.578) and VAS flexion (*r* = 0.529) showed a large effect size. Table 2 shows the results regarding the effect sizes.

#### Within-group analysis

The longitudinal analyses showed statistically significant differences between time in both groups and in every individual variable (Friedman’s analysis of variance, *p* < 0.05). Wilcoxon signed rank tests revealed that WOMAC total scores, WOMAC pain, WOMAC disability, EQ-5D-5L, 30 CST, active knee extension and VAS rest all steadily improved over time until 6 months in the low PCS group. On the other hand, this improvement decelerated at 3 months in the high PCS group, showing a lack of significance between 3 and 6 months. These findings were not observed in WOMAC stiffness, 4MWT, active knee flexion, VAS walking or VAS flexion (see Table 3).

**Table 2:** Outcome measures between groups.

**Outcome measures**	**Median (first and third quartiles)**		***U***	***Z***	***p***	***r***
	**Low PCS (<14.5)**	**High PCS (14.5)**				
WOMAC (0–96 range)						
1 week	29 (21 and 45)	50 (41.75 and 65.29)	155	−4.363	.000	.563^c^
1 month	20.5 (13.75 and 26.5)	34 (23.75 and 53.25)	200	−3.699	.000	.578^c^
3 months	15 (7.75 and 26)	30 (11.50 and 46.25)	267.5	−2.700	.007	.349^b^
6 months	9 (5 and 19)	28.5 (8.50 and 39)	210	−3.416	.001	.445^b^
WOMAC pain (0–20 range)						
1 week	5.5 (3 and 7)	9 (7.38 and 11.44)	125.5	−4.820	.000	.622^c^
1 month	4 (2 and 6)	7 (4.75 and 10.25)	207.5	−3.602	.000	.465^b^
3 months	3 (1 and 5)	5.5 (2 and 10.25)	296	−2.288	.022	.295^a^
6 months	2 (1 and 3)	6 (2 and 8.25)	228.5	−3.157	.002	.411^b^
WOMAC stiffness (0–8 range)						
1 week	2.5 (2 and 4)	4 (3 and 5.25)	202.5	−3.737	.000	.482^b^
1 month	2 (1 and 3)	3.5 (3 and 4.25	236.5	−3.222	.001	.416^b^
3 months	1.5 (1 and 3)	3 (1 and 4.25)	317.5	−1.999	.046	.258^a^
6 months	1 (0 and 1.5)	2 (.75 and 4)	269.5	−2.596	.009	.338^b^
WOMAC disability (0–68 range)						
1 week	21.5 (16 and 34.5)	37.20 (30.75 and 50)	178	−4.023	.000	.519^c^
1 month	15 (9.75 and 19)	23.50 (16.25 and 36)	213.5	−3.501	.000	.452^b^
3 months	11.5 (4.75 and 18.25)	22 (7.75 and 30)	262	−2.782	.005	.359^b^
6 months	5 (3.50 and 14)	19 (6.50 and 27.5)	198	−3.601	.000	.469^b^
EQ–5D–5L (0–1 range)						
1 week	.608 (.527 and .527)	.281 (.076 and .480)	131.5	−4.710	.000	.608^c^
1 month	.728 (.636 and .849)	.648 (.354 and .748)	276.5	−2.668	.010	.332^b^
3 months	.849 (.742 and .910)	.721 (.590 and .843)	289.5	−2.379	.017	.307^b^
6 months	.910 (.412 and .759)	.709 (.562 and .897)	222	−3.258	.001	.424^b^
4MWT (meters/second)						
1 week	.599 (.412 and .759)	.413 (.309 and .727)	308	−2.100	.036	.271^a^
1 month	.812 (.676 and 1.063)	.780 (.553 and 1.002)	390.5	−.880	.379	.114^a^
3 months	1.127 (.889 and 1.194)	.920 (.791 and 1.022)	286.5	−2.417	.016	.312^b^
6 months	1.226 (.949 and 1.434)	1.041 (.791 and 1.167)	321	−1.728	.084	.225^a^
30s CST (repetitions)						
1 week	6 (2.25 and 9)	0.5 (.00 and 5.25)	255	−2.965	.003	.284^a^
1 month	9.5 (6 and 12.25)	7 (4.75 and 10)	302	−2.196	.028	.284^a^
3 months	12 (9 and 14)	9 (6 and 11.25)	258.5	−2.844	.004	.367^b^
6 months	12 (9 and 15)	12 (9 and 15)	248.5	−2.838	.005	.369^b^
Active knee flexion (degrees)						
1 week	85 (70 and 95)	80 (65 and 90)	376	−1.100	.271	.142^a^
1 month	102.5 (90 and 110)	100 (90 and 110)	439	−.164	.870	.021
3 months	110 (100 and 116.25)	110 (100 and 116.25)	446.5	−.052	.958	.007
6 months	115 (110 and 122.5)	110 (105 and 125)	403.5	−.482	.630	.063
Active knee extension (degrees)						
1 week	−10 (−11.25 and −8.75)	−10 (−15 and −10)	385.5	−1.043	.279	.135^a^
1 month	−10 (−10 and −5)	−10 (−10 and −5)	406	−0.701	.483	.090
3 months	−5 (−5 and 0)	−5 (−10 and −5)	347	−1.646	.100	.212^a^
6 months	0 (−5 and 0)	−5 (−5 and 0)	310.5	−2.063	.039	.269^a^
VAS Rest (0–10 range)						
1 week	3 (1.375 and 4)	5.75 (4 and 7)	147.5	−4.502	.000	.581^c^
1 month	2 (.75 and 3.25)	4 (2 and 7)	252.5	−2.948	.003	.381^b^
3 months	0 (0 and 2)	3.5 (0 and 6)	267.5	−2.824	.005	.365^b^
6 months	0 (0 and 1.5)	2 (0 and 5)	230.5	−3.255	.001	.424^b^
VAS Walking (0–10 range)						
1 week	3 (1 and 4.125)	6 (4.75 and 8)	151	−4.438	.000	.573^c^
1 month	2 (1 and 4)	5 (2.75 and 7)	231.5	−3.249	.001	.419^b^
3 months	3 (0 and 5)	4.5 (2 and 6.25)	319	−1.954	.051	.252^a^
6 months	2 (0 and 3.25)	3 (1.75 and 6)	258.5	−2.703	.007	.352^b^
VAS Flexion (0–10 range)						
1 week	6 (3 and 7)	8 (7 and 9)	175.5	−4.101	.000	.529^c^
1 month	3 (2 and 6)	7 (5 and 9)	174.5	−4.096	.000	.529^c^
3 months	3.25 (.75 and 5)	5 (3 and 8)	244	−3.066	.002	.396^b^
6 months	2 (0 and 3.5)	3.75 (2 and 6)	251.5	−2.802	.005	.365^b^

**Notes.**

PCS Pain Catastrophizing Scale WOMAC Western Ontario and McMaster Osteoarthritis Index 5Q-5D-5L Euro Quality of Life 5 Dimensions—5 Levels 4MWT 4 Meters Walking Test 30s CST 30-second Chair Stand Test VAS Visual Analog Scale U Mann Whitney’s U r Effect size

a Small effect size 0.1–0.29.

b Medium effect size 0.3–0.49.

c Large effect size > 0.5.

**Table 3:** Outcome measures within groups.

	**Group**	**Friedman ANOVA**	**Wilcoxon**
WOMAC (0–96 range)	Low PCS	.000	.000^a^, .000^b^, .000^c^, .027^d^, .000^e^, .001^f^
	High PCS	.000	.000^a^, .000^b^, .000^c^, .009^d^, .001^e^, .092^f^
WOMAC Pain (0–20 range)	Low PCS	.000	.003^a^, .000^b^, .000^c^, .207^d^, .001^e^, .005^f^
	High PCS	.000	.001^a^, .000^b^, .000^c^, .028^d^, .001^e^, .262^f^
WOMAC Stiffness (0–8 range)	Low PCS	.000	.073^a^, .004^b^, .000^c^, .182^d^, .000^e^, .001^f^
	High PCS	.000	.023^a^, .001^b^, .000^c^, .015^d^, .000^e^, .050^f^
WOMAC Disability (0–68 range)	Low PCS	.000	.000^a^, .000^b^, .000^c^, .014^d^, .000^e^, .003^f^
	High PCS	.000	.000^a^, .000^b^, 000^c^, .007^d^, .001^e^, .190^f^
EQ-5D-5L (0–1 range)	Low PCS	.000	.000^a^, .000^b^, .000^c^, .004^d^, .000^e^, .023^f^
	High PCS	.000	.000^a^, .000^b^, .000^c^, .002^d^, .004^e^, .914^f^
4MWT (meters/second)	Low PCS	.000	.000^a^, .000^b^, .000^c^, .000^d^, .000^e^, .010^f^
	High PCS	.000	.000^a^, .000^b^, .000^c^, .000^d^, .000^e^, .001^f^
30s CST (repetitions)	Low PCS	.000	.000^a^, .000^b^, .000^c^, .000^d^, .000^e^, .076^f^
	High PCS	.000	.000^a^, .000^b^, .000^c^, .002^d^, .003^e^, .341^f^
Active Knee Flexion (degrees)	Low PCS	.000	.000^a^, .000^b^, .000^c^, .000^d^, .000^e^, .011^f^
	High PCS	.000	.000^a^, .000^b^, .000^c^, .000^d^, .000^e^, .008^f^
Active Knee Extension (degrees)	Low PCS	.000	.001^a^, .000^b^, .000^c^, .003^d^, .000^e^, .037^f^
	High PCS	.000	.001^a^, .000^b^, .000^c^, .019^d^, .000^e^, .062^f^
VAS Rest (0–10 range)	Low PCS	.000	.015^a^, .000^b^, .000^c^, .004^d^, .000^e^, .061^f^
	High PCS	.000	.007^a^, .000^b^, .000^c^, .013^d^, .001^e^, .552^f^
VAS Walking (0–10 range)	Low PCS	.042	.452^a^, .736^b^, .025^c^, .653^d^, .050^e^, .029^f^
	High PCS	.000	.010^a^, .000^b^, .000^c^, .026^d^, .001^e^, .239^f^
VAS Flexion (0–10 range)	Low PCS	.000	.007^a^, .001^b^, .000^c^, .055^d^, .002^e^, .023^f^
	High PCS	.000	.002^a^, .000^b^, .000^c^, .002^d^, .000^e^, .004^f^

**Notes.**

PCS Pain Catastrophizing Scale WOMAC Western Ontario and McMaster Osteoarthritis Index 5Q-5D-5L Euro Quality of Life 5 Dimension—5 Levels 4MWT 4 Meters Walking Test 30s CST 30-second Chair Stand Test VAS Visual Analog Scale

a 1 week vs. 1 month.

b 1 week vs. 3 months.

c 1 week vs. 6 months.

d 1 month vs. 3 months.

e 1 month vs. 6 months.

f 3 months vs. 6 months.

## Discussion

The present study aimed to investigate the postoperative health functioning on subjects who underwent KA due to OA in relation to postoperative pain catastrophizing. Further, this research aimed to study their quality of life, pain, walking speed, physical performance, and ROM in relation to pain catastrophizing. The findings suggest that subjects with higher early postoperative pain catastrophizing had worse postoperative outcomes after KA.

In concordance with previous studies, pain catastrophizing has shown to be highly correlated with pain intensity after KA (Wade, Riddle & Thacker, 2012). These findings suggest that individuals are more likely to catastrophize when pain is more intense. Therefore, it is possible that the group with higher PCS levels did not truly capture highly catastrophizing subjects. Nevertheless, using PCS as a grouping variable did show interesting differences between groups which are worth to be discussed.

### PCS and health functioning

To our knowledge, there are no previous studies that have analyzed the relationship between the early postoperative PCS and health functioning outcomes during the rehabilitation period after KA. Nevertheless, some studies have used preoperative PCS levels, which we considered relevant for discussion. In this regard, the current evidence concerning the influence of PCS in postoperative health functioning is conflicting. Yang et al. (2019) found that participants with a suboptimal function-improvement trajectory following total KA had higher PCS compared with those with an optimal trajectory. Similarly, Bierke & Petersen (2017) observed that pain catastrophizing had a significant effect on knee function 6 months preoperatively, and according to Sullivan et al. (2011), pain catastrophizing predicted poorer recovery from total KA. On the other hand, Riddle et al. (2010) found that PCS might not be a predictor of function outcome. Along these lines, Mark-Christensen & Kehlet (2019) observed that participants with low physical functioning who were scheduled for a total KA had slightly higher levels of PCS both preoperatively and 4 months postoperatively. However, no significant differences were observed between the groups, and no strong correlations were observed between preoperative PCS and function. In the present study, the participants with higher levels of early postoperative PCS achieved poorer results in WOMAC disability scores and WOMAC total score at each time point. In addition, health functioning improved in both groups during all rehabilitation processes, but in the high PCS group, this improvement stopped at the 3-month follow-up. These results might suggest that participants in the high pain catastrophizing group are at greater risk of reduced functional improvements after KA. Future studies with proper analysis should be performed in order to investigate this relationship.

Catastrophic thinking has been shown to interfere with fundamental neural processes related to pain perception through excessive attention, anticipation and heightened emotional responses to pain, making it more challenging to shift the focus of attention away from threatening stimuli (Gracely et al., 2004). According to the fear-avoidance model, this leads to avoidance behaviors and hypervigilance to bodily sensations, followed by disability, disuse and depression (Vlaeyen & Linton, 2012). In fact, it has been observed that in participants with OA, pain catastrophizing might be an obstacle to participants’ willingness to engage in demanding physical activities (e.g., walking fast), even though such activities are essential in managing pain and disability (Somers et al., 2009). Therefore, interventions aiming to address and modify such cognitions might enable patients to feel more confident about engaging in activities and beginning them earlier after the surgery. By becoming more confident, in physiotherapy interventions to increase activity despite pain, KA patients might be able to start moving earlier, reduce their postoperative pain, and improve their functioning faster.

### PCS and postoperative outcomes

The impact of preoperative pain catastrophizing on persistent postsurgical pain has been thoroughly studied during the last decade. According to a systematic review with meta-analysis performed by Lewis et al. (2015), catastrophizing emerged as one of the most relevant predictors of persistent postsurgical pain. In line with these results, we found that participants with high postoperative PCS had greater levels of pain intensity at every time point. In addition, as expected, there was a significant decrease in pain scores after KA, but it varied between groups. Pain reductions decelerated at the 3-month follow-up in the high PCS group, whereas they continued in the low PCS group during the entire 6-month rehabilitation period. Given the study design, no conclusions in that regard can be drawn. Therefore, future studies should be performed in order to investigate whether early postoperative pain catastrophizing plays a significant role in pain reduction during the rehabilitation process after KA.

Psychosocial variables such as pain catastrophizing have been shown to be more influential in the development of chronic pain after KA than function, depression, comorbidities or pain intensity (Lewis et al., 2015). High catastrophizing patients’ tendency to focus excessively on pain sensations has been discussed as a mechanism that contributes to altered central thresholds of excitability or to the amplification of pain signals. Catastrophizing has been shown to influence pain perception directly, through its influence on affective and attentional responses to pain (Gracely et al., 2004). Consequently, some authors suggest that interventions designed to shift attention to the perceived threat of clinical pain might be beneficial for those with pain who catastrophize about their condition (Yakobov et al., 2014; Burns et al., 2015; Buvanendran et al., 2019).

Despite that, Lee et al. (2016) investigated whether the change in pain catastrophizing would mediate between an initial improvement in pain biology knowledge and subsequent reduction in pain and improvement in function. Their findings showed that despite pain catastrophizing reduction after a pain neuroscience education intervention, changes in catastrophizing did not mediate the effect of pain knowledge acquisition in pain or function improvements (Lee et al., 2016). These results suggest that pain catastrophizing might not be a fruitful target for intervention. Nevertheless, this study was performed on unspecific chronic musculoskeletal pain treatment, so it may be different in postoperative settings. Several studies suggest that targeting PCS could be an effective preventative intervention in subjects scheduled for KA (Sullivan, Bishop & Pivik, 2009; Riddle et al., 2010; Wideman & Sullivan, 2011; Bierke & Petersen, 2017; Hirakawa et al., 2014). Also, Sullivan, Bishop & Pivik (2009) highlighted the importance of the previous examination for at-risk patients in developing poor outcomes after KA. If individuals at-risk for post-surgical poor results can be identified before the problem becomes chronic, the individual’s suffering might be prevented or reduced to a significant degree (Sullivan, Bishop & Pivik, 2009). Therefore, it would be interesting to investigate the effectiveness of preventative interventions, specifically targeting pain catastrophizing in high PCS subjects scheduled for KA.

Preoperative pain catastrophizing has also been associated with HRQoL after KA. This association could be explained by the negative influence that activity avoidance exerts on HRQoL by depriving the individual of participating in important life and social activities. Furthermore, pain catastrophizing has a direct influence on pain intensity (Hadlandsmyth et al., 2017), mental health and fundamental functional abilities such as walking, which directly affect HRQoL (Yakobov et al., 2018). Our results showed that participants with high postoperative PCS had lower HRQoL during all rehabilitation periods and had less significant improvements over time when compared with those with low PCS.

Walking speed and physical performance are two relevant variables for participants with KA, with a strong correlation between them (Unver et al., 2015). Despite a lack of evidence from other studies, speed and performance were also found to be lower in the high PCS group. As noted earlier, catastrophic thinking can dissuade individuals with OA from performing demanding physical activities. Furthermore, it can lead them to develop avoidance behaviors affecting their walking speed and physical performance (Somers et al., 2009). In addition, pain catastrophizing has been found to influence pain intensity via muscle weakness and disability (Tanaka, Hirohama & Ozawa, 2019), and our results showed that those with high PCS had increased pain, muscle weakness, and disability, suggesting this relationship. In line with these results, Somers et al. (2009) also found that pain catastrophizing affected walking speed in participants with OA, and Mark-Christensen & Kehlet (2019) observed that participants with poorer physical performance had significantly greater levels of pain catastrophizing.

Finally, and according to previous studies, there were no significant differences between groups in ROM at any follow-up. Mann et al. (2019) also found no significant differences in PCS total score when comparing participants with and without knee stiffness. In this research, knee stiffness was defined as either a failure to achieve 90° of flexion and/or a total ROM of less than 90° at 6 weeks postoperatively. Postoperative knee stiffness has been shown to be a multifactorial problem, and biological factors appear to play a significant role in their development while the influence of psychosocial factors remains unclear (Mann et al., 2019).

### Limitations and strengths

This study has several limitations that should be considered when interpreting the results:

- The project’s aim was modified from the initial protocol.
- No statistical plan was provided in the protocol; therefore, this study has been performed as a hypothesis-generating study. Due to the study design, no sample size calculation was conducted, and because of the small sample, no univariate or multivariate regression analysis was performed.
- In order to investigate the associations between PCS and postoperative outcomes, the cohort was divided into two groups based on their PCS total score (50th percentile cut-off).
- The discussion proceeded from the perspective that postoperative PCS contributes to poorer outcomes, and it is very likely that this association is bi-directional; thus, it might be possible that poor outcomes could also lead to heightened pain catastrophizing. Consequently, pain intensity should be considered as a potential confounder when assessing PCS for a causal relationship with postoperative outcomes. Therefore, in this study, no causal relationships can be done between PCS and postoperative outcomes. Future research with higher sample size and proper statistical analysis should be done.
- Despite the baseline characteristics analysis, postoperative outcomes might have been influenced by other factors not measured in this study.
- Variables such as mental health, social support, or patient expectations may influence postoperative outcomes as well and should be taken into account in future studies (Sullivan et al., 2011; Zeppieri et al., 2019).
- Participants with negative emotions, such as anxiety or depression, might produce negative responses, resulting in negativity bias.
- Subjects were admitted to rehabilitation service one week after surgery; for that reason, subjects were not assessed before surgery or immediately after surgery. Therefore, relevant information about the preoperative PCS association with postoperative outcomes was not available.
- Although the exploratory nature of the study, the statistical analysis did not consider the possible inflation of error rates due to multiple testing.

As a consequence of all these limitations, the results of the present study should be interpreted with caution.

As a result of performing all the assessments at the participants’ homes, the dropout rate was meager when compared with the percentages of other studies evaluating the impact of psychosocial factors on KA outcomes. Dropouts in longitudinal studies are frequent and entail a potential source of bias, therefore it should be considered as a strength.

### Clinical implications and future studies

Collectively, these findings suggest that postoperative PCS could play a role during the subjects’ rehabilitation process. Therefore, future studies should investigate if there is a causal relationship between postoperative PCS and KA outcomes. These studies should include additional psychosocial variables, such as pain-related fear of movement, self-efficacy, social support, or expectancies, which could also influence the outcomes (Van den Akker-Scheek et al., 2007; Sullivan et al., 2011; Wylde et al., 2017; Zeppieri et al., 2019).

Despite the growing evidence of psychosocial factors’ significance in this population, they are not usually considered in rehabilitation after KA. Physiotherapy interventions after KA have mainly focused on improving physical outcomes (e.g., physical function or ROM), and they have only shown short-term benefits (Artz et al., 2015). Therefore, several authors suggest that targeted interventions to reduce pain catastrophizing might be a more effective approach for high PCS participants (Sullivan, Bishop & Pivik, 2009; Riddle et al., 2010; Wideman & Sullivan, 2011; Bierke & Petersen, 2017).

Pain catastrophizing has been shown to be modifiable with interventions. Biobehavioral interventions such as pain neuroscience education or cognitive-behavioral therapy have been shown to reduce PCS levels in various pain conditions (Smeets et al., 2006; Louw et al., 2016). Consequently, future studies are needed to investigate whether screening and managing pain catastrophizing can improve postoperative outcomes after KA.

## Conclusion

The results of this study suggest that participants with high postoperative pain catastrophizing might have poorer postoperative health outcomes during the rehabilitation process after a KA. Future work should seek to clarify if this relationship is causal.

##  Supplemental Information

10.7717/peerj.9903/supp-1 Supplemental Information 1 Supplementary Figures

10.7717/peerj.9903/supp-2 Supplemental Information 2 Raw data

## Additional Information and Declarations

### Data Availability

The following information was supplied regarding data availability: The raw data are available in the Supplemental File.
