# Supplementary material for: Assessment of postoperative health functioning after knee arthroplasty in relation to pain catastrophizing: a 6-month follow-up cohort study
Source: PeerJ. 2020 Sep 9;8:e9903. doi: 10.7717/peerj.9903 (PMC7486825; doi:10.7717/peerj.9903)
Supplement: Supplemental Information 1 [file peerj-08-9903-s001.pdf]

Supplementary Information

Assessment of postoperative health functioning after knee arthroplasty in relation to pain catastrophizing: A 6-month follow-up cohort study

Marc Terradas-Monllor, Mirari Ochandorena-Acha, Julio Salinas-Chesa, Sergi Ramirez-Ramos, Hector Beltran-Alacreu

Supplementary Figures

Figure S1	WOMAC Total Score differences between groups during rehabilitation process.
Figure S2	WOMAC Pain differences between groups during rehabilitation process.
Figure S3	WOMAC Stiffness differences between groups during rehabilitation process.
Figure S4	WOMAC disability differences between groups during rehabilitation process.
Figure S5	EQ-5D-5L differences between groups during rehabilitation process.
Figure S6	4 Meters Walking Test differences between groups during rehabilitation process.
Figure S7	30-second Chair Stand Test differences between groups during rehabilitation process.
Figure S8	Active Knee Flexion differences between groups during rehabilitation process.
Figure S9	Active Knee Extension differences between groups during rehabilitation process.
Figure S10	VAS Rest differences between groups during rehabilitation process.
Figure S11	VAS Walking differences between groups during rehabilitation process.
Figure S12	VAS Flexion differences between groups during rehabilitation process.

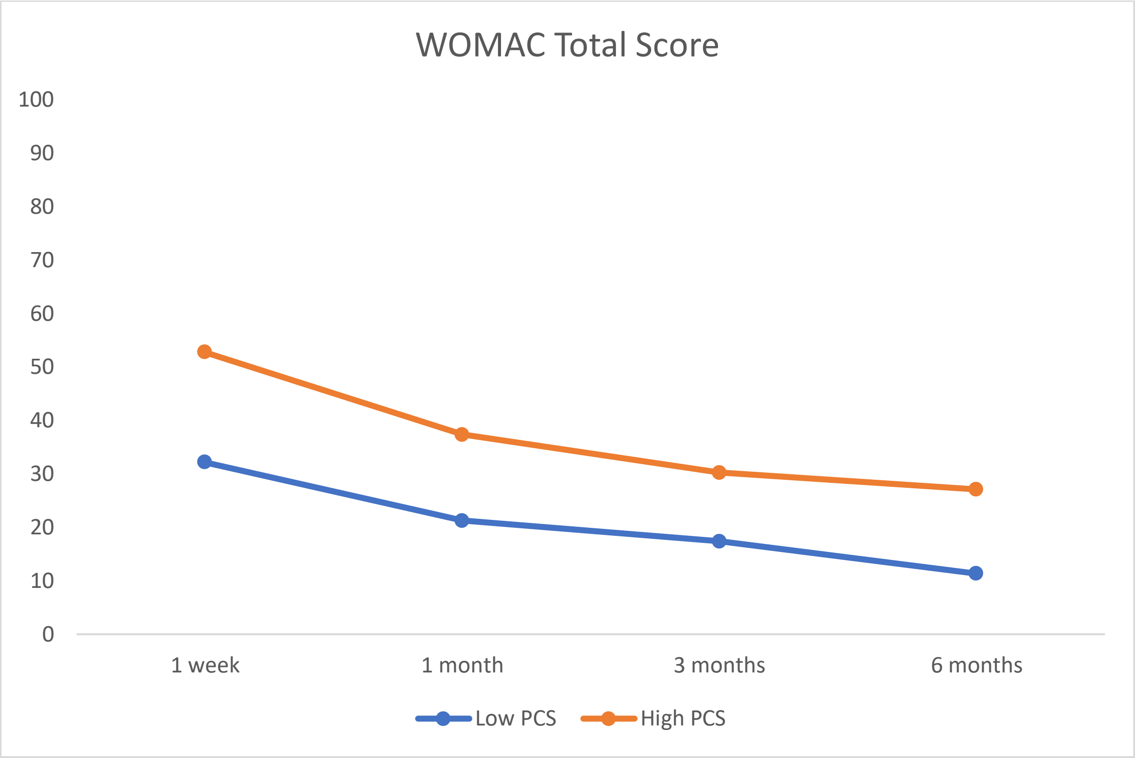

Figure S1. WOMAC Total Score differences between groups during rehabilitation process.
WOMAC = Western Ontario and McMaster Osteoarthritis Index.

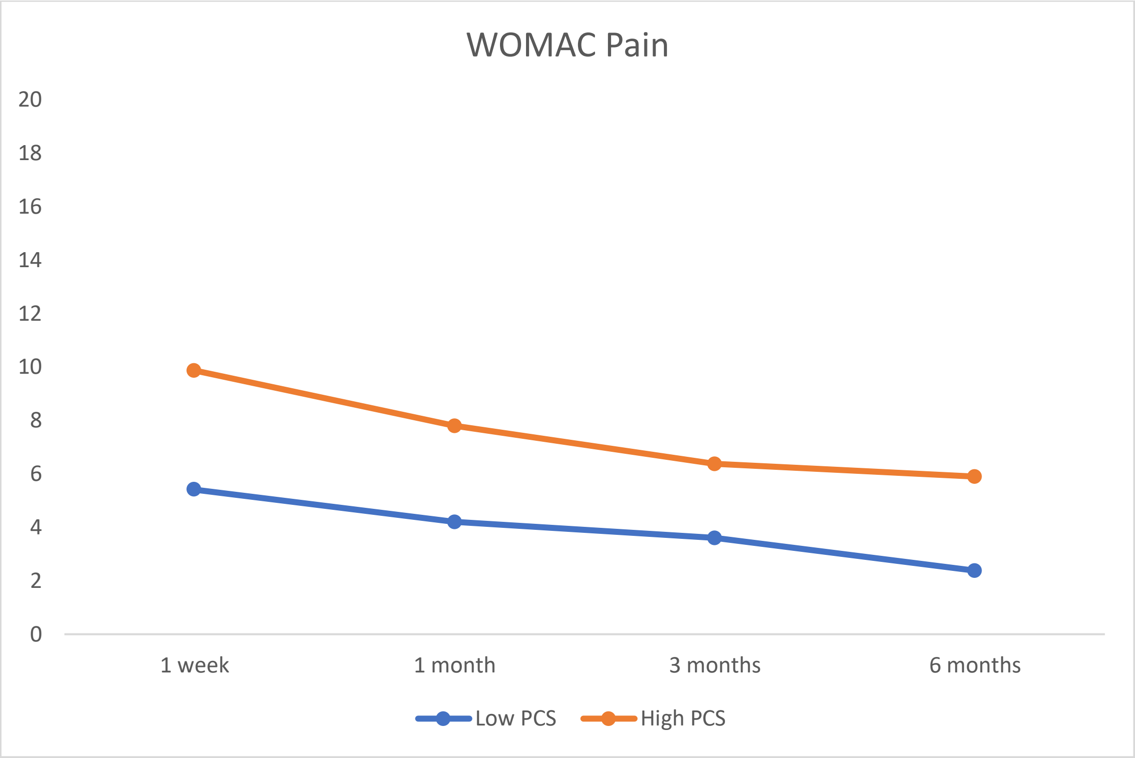

Figure S2. WOMAC Pain differences between groups during rehabilitation process.

WOMAC = Western Ontario and McMaster Osteoarthritis Index.

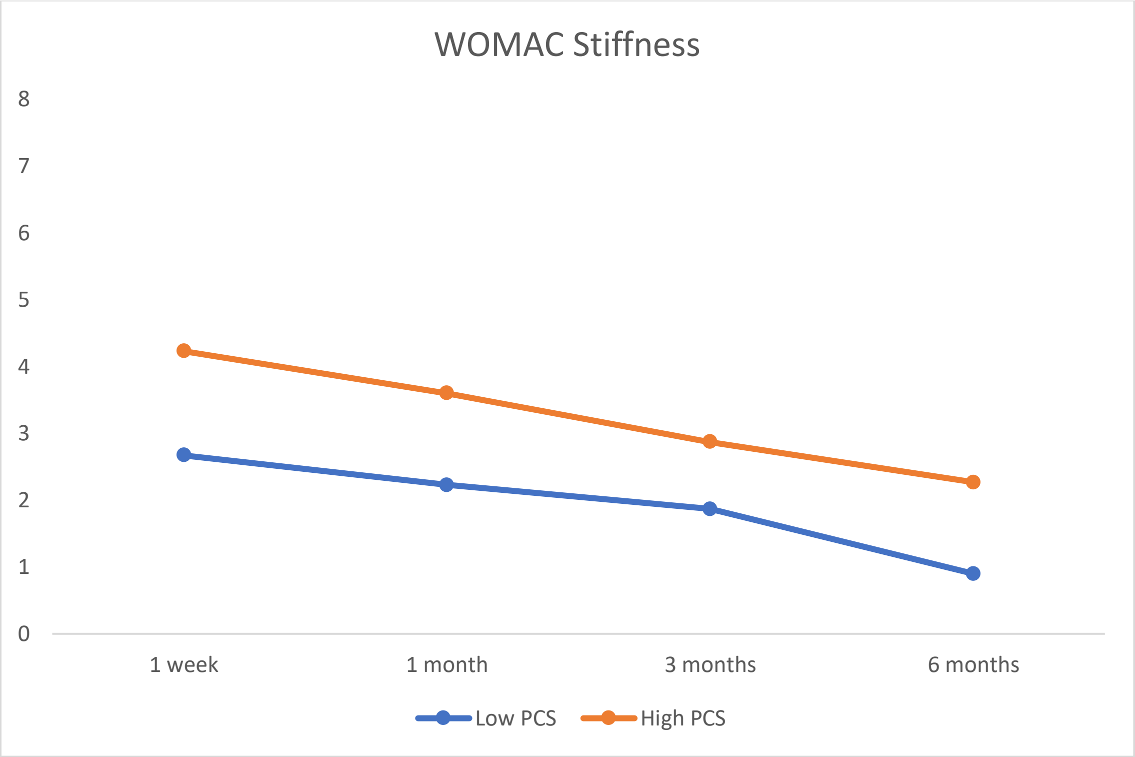

Figure S3. WOMAC Stiffness differences between groups during rehabilitation process. WOMAC = Western Ontario and McMaster Osteoarthritis Index.

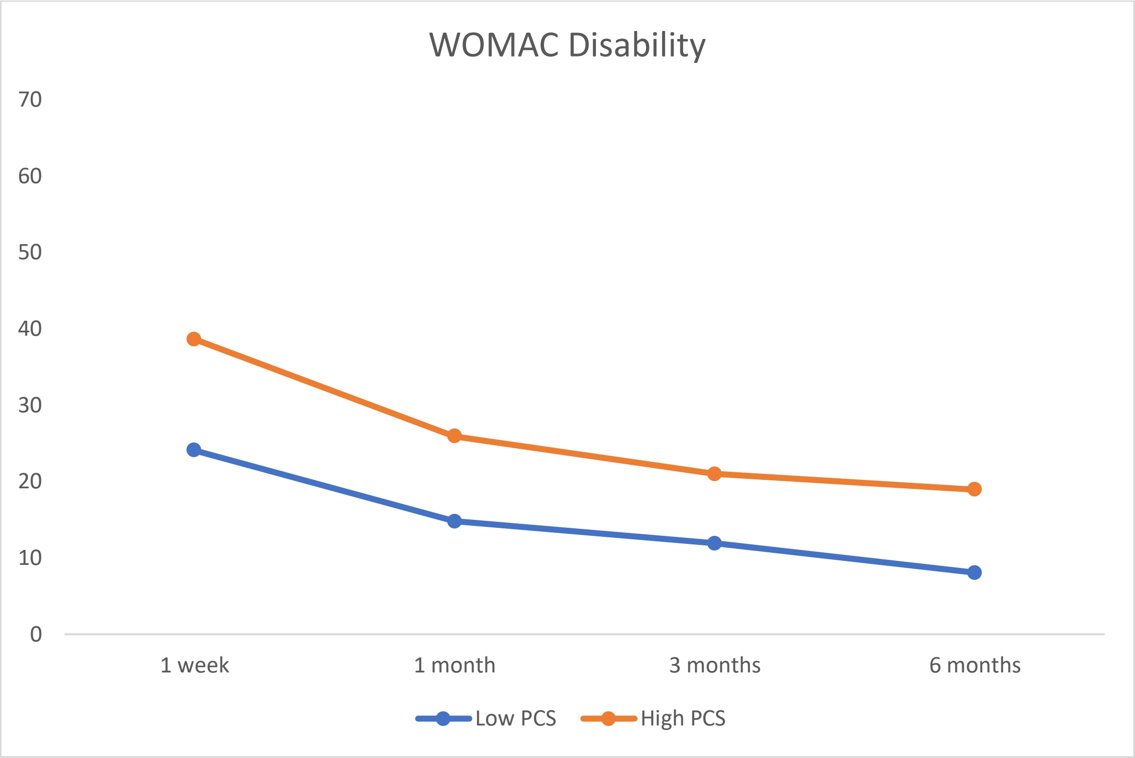

Figure S4. WOMAC Disability differences between groups during rehabilitation process. WOMAC = Western Ontario and McMaster Osteoarthritis Index.

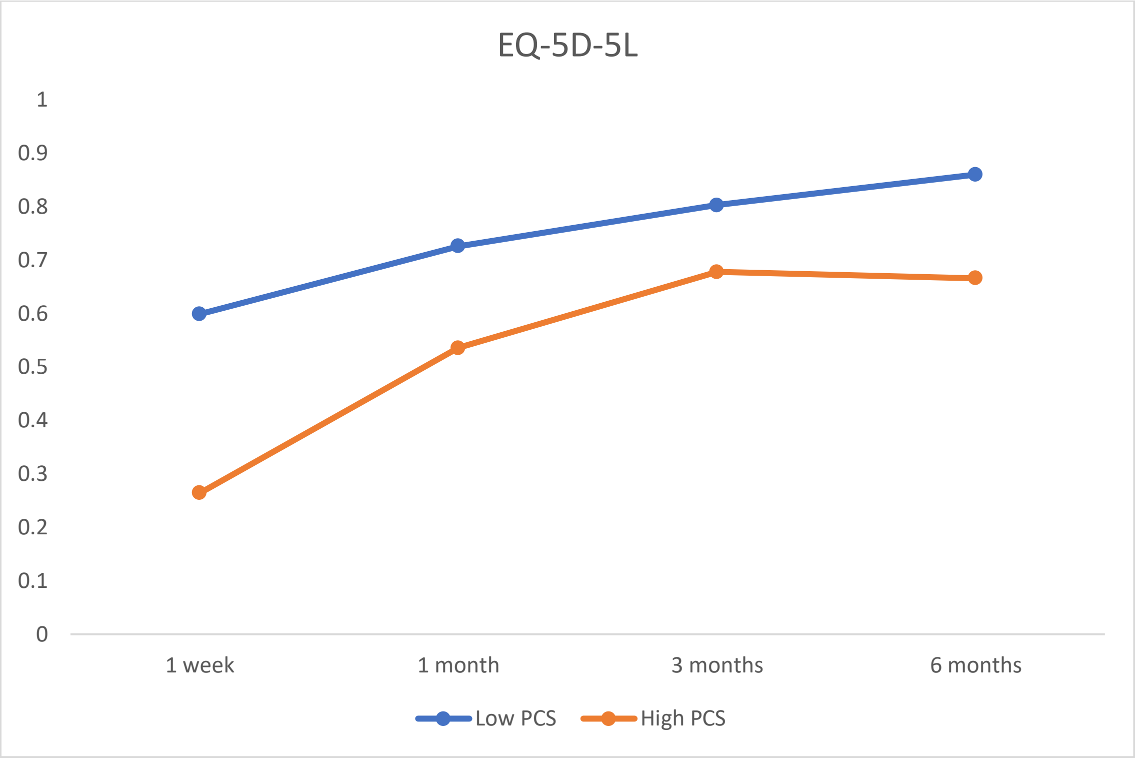

Figure S5. EQ-5D-5L differences between groups during rehabilitation process.

EQ-5D-5L = Euro Quality of Life 5 Dimensions – 5 Levels.

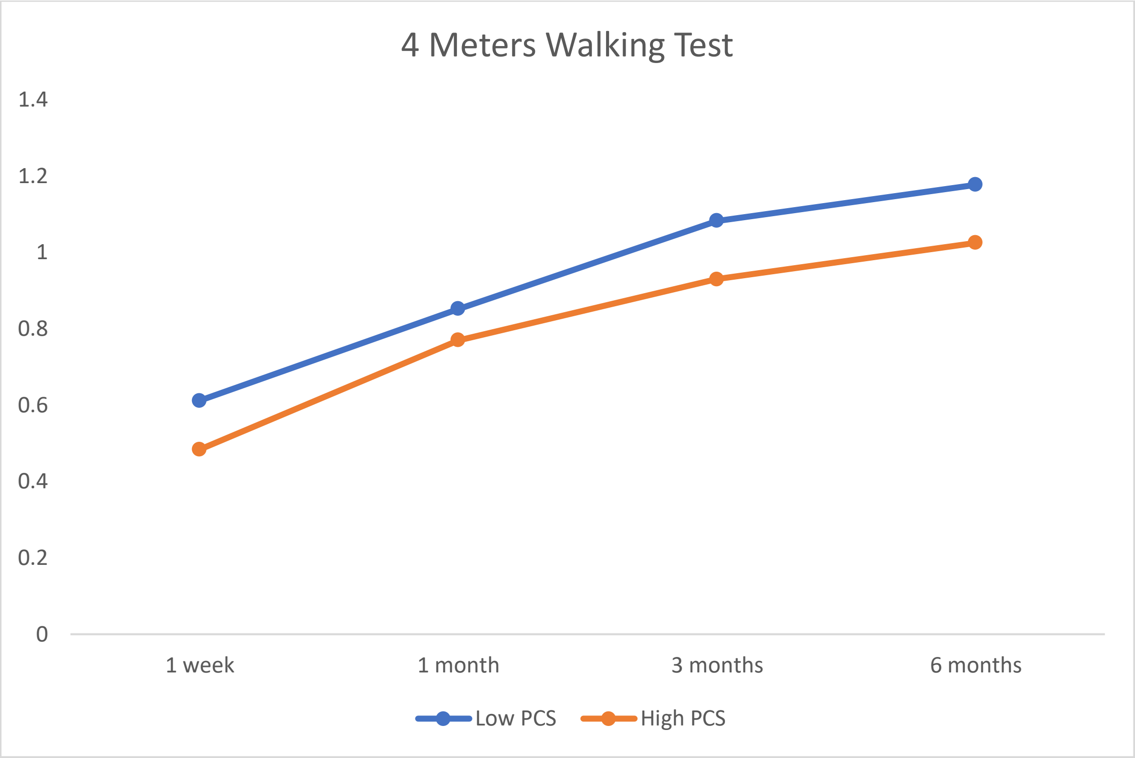

Figure S6. 4 Meters Walking Test differences between groups during rehabilitation process.

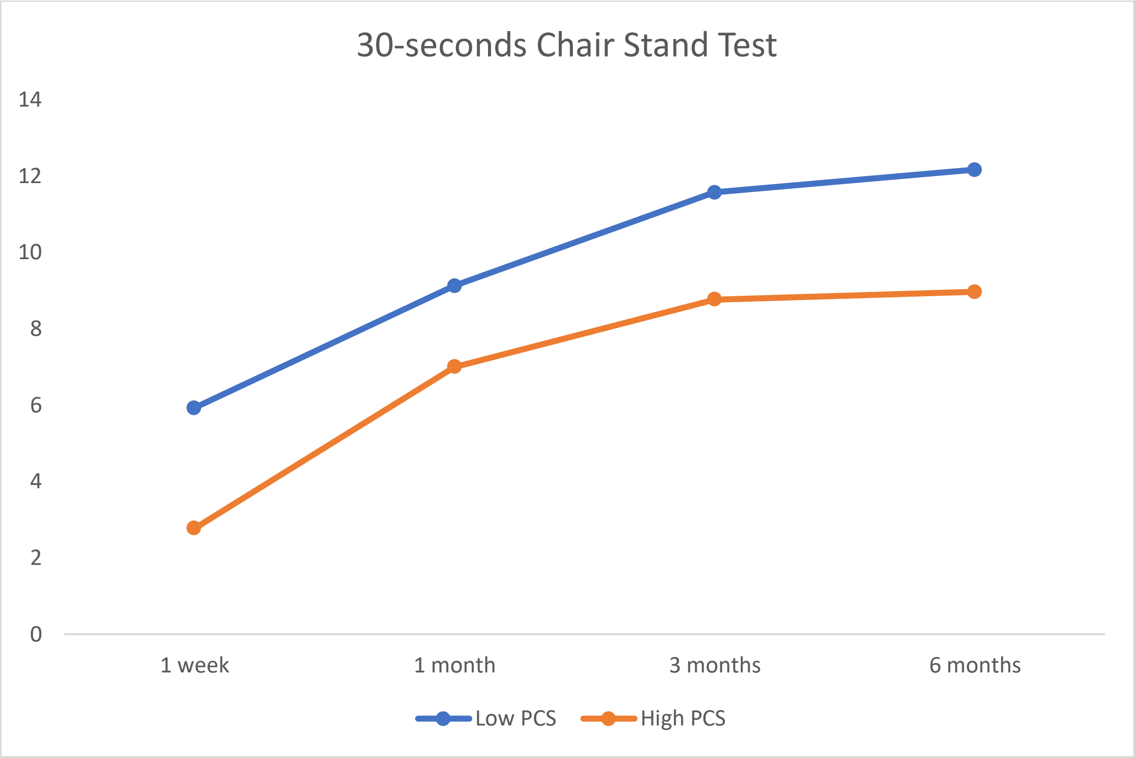

Figure S7. 30-second Chair Stand Test differences between groups during rehabilitation process.

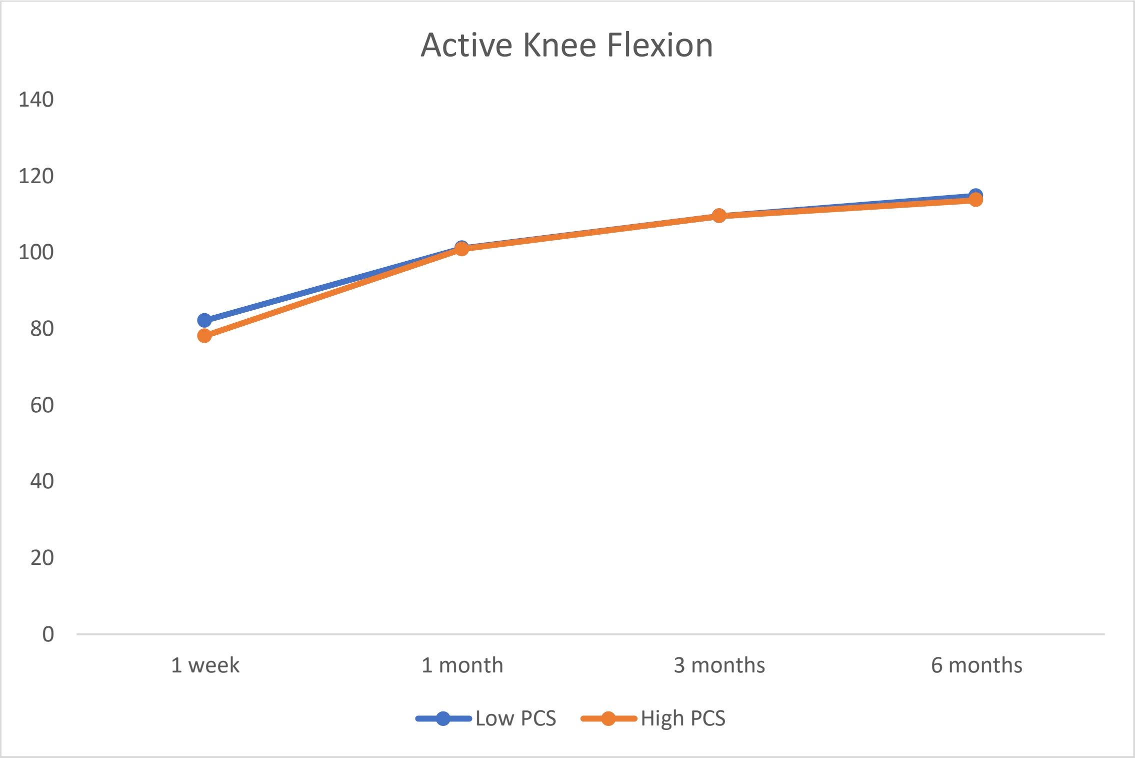

Figure S8. Active Knee Flexion differences between groups during rehabilitation process.

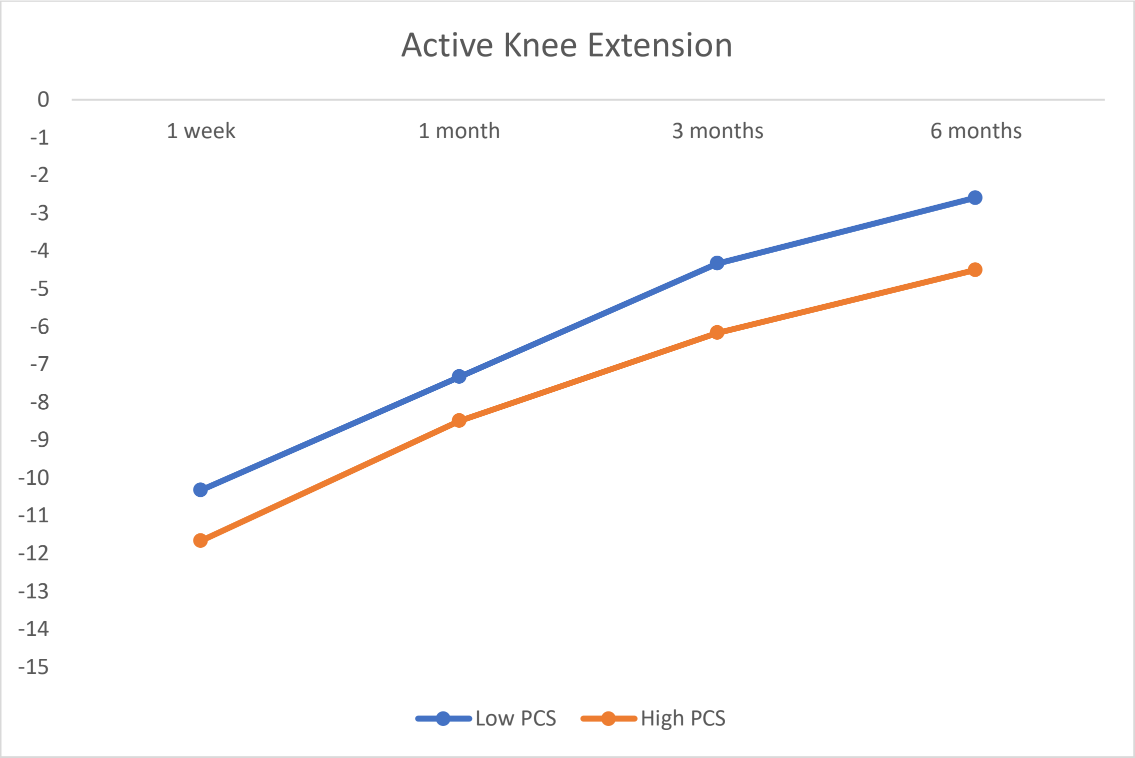

Figure S9. Active Knee Extension differences between groups during rehabilitation process.

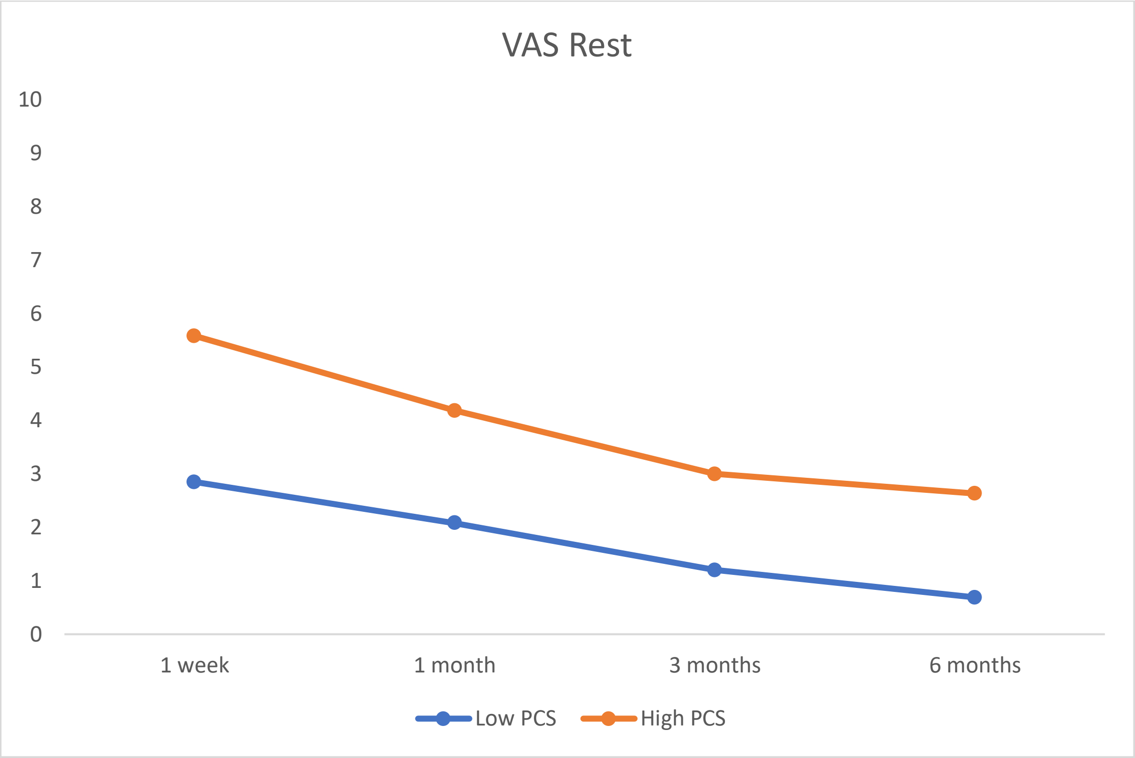

Figure S10. VAS Rest differences between groups during rehabilitation process.

VAS = Visual Analog Scale

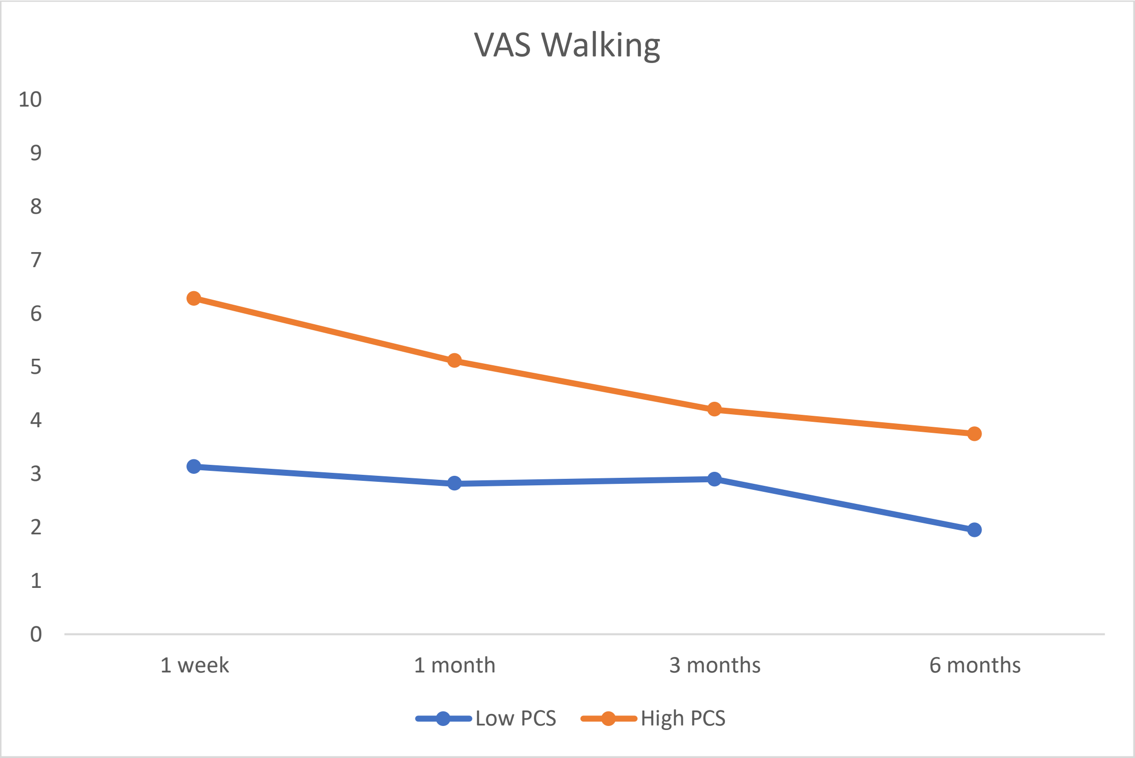

Figure S11. VAS Walking differences between groups during rehabilitation process.

VAS = Visual Analog Scale

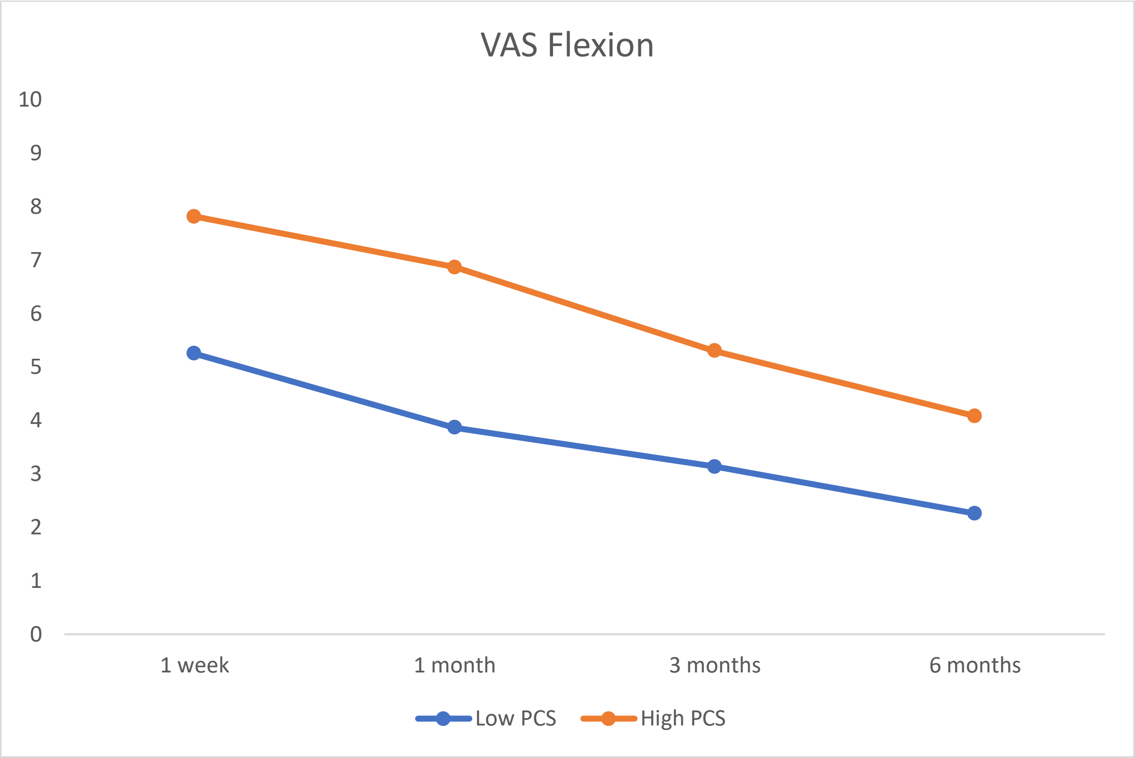

Figure S12. VAS Flexion differences between groups during rehabilitation process.

VAS = Visual Analog Scale
